# Extreme Fire Severity Patterns in Topographic, Convective and Wind-Driven Historical Wildfires of Mediterranean Pine Forests

**DOI:** 10.1371/journal.pone.0085127

**Published:** 2014-01-22

**Authors:** Judit Lecina-Diaz, Albert Alvarez, Javier Retana

**Affiliations:** 1 CREAF, Cerdanyola del Vallès, Catalonia, Spain; 2 Departament de Biologia Animal, Biologia Vegetal i Ecologia, Universitat Autònoma de Barcelona, Cerdanyola del Vallès, Catalonia, Spain; Lakehead University, Canada

## Abstract

Crown fires associated with extreme fire severity are extremely difficult to control. We have assessed fire severity using differenced Normalized Burn Ratio (dNBR) from Landsat imagery in 15 historical wildfires of *Pinus halepensis* Mill. We have considered a wide range of innovative topographic, fuel and fire behavior variables with the purposes of (1) determining the variables that influence fire severity patterns among fires (considering the 15 wildfires together) and (2) ascertaining whether different variables affect extreme fire severity within the three fire types (topographic, convective and wind-driven fires). The among-fires analysis showed that fires in less arid climates and with steeper slopes had more extreme severity. In less arid conditions there was more crown fuel accumulation and closer forest structures, promoting high vertical and horizontal fuel continuity and extreme fire severity. The analyses carried out for each fire separately (within fires) showed more extreme fire severity in areas in northern aspects, with steeper slopes, with high crown biomass and in climates with more water availability. In northern aspects solar radiation was lower and fuels had less water limitation to growth which, combined with steeper slopes, produced more extreme severity. In topographic fires there was more extreme severity in northern aspects with steeper slopes and in areas with more water availability and high crown biomass; in convection-dominated fires there was also more extreme fire severity in northern aspects with high biomass; while in wind-driven fires there was only a slight interaction between biomass and water availability. This latter pattern could be related to the fact that wind-driven fires spread with high wind speed, which could have minimized the effect of other variables. In the future, and as a consequence of climate change, new zones with high crown biomass accumulated in non-common drought areas will be available to burn as extreme severity wildfires.

## Introduction

Forest fires are common in many parts of the world, including the Mediterranean ecosystems [1]. Depending on the strata burnt, wildfires could be classified into ground, surface and crown fires [2] . Crown fires are those that burn in elevated canopy fuels, which mainly include active crown fires, when fuel and weather allow fire to spread continuously between tree crowns involving the entire surface-canopy complex, and passive crown fires, when one tree or a group of trees burns individually without a solid flame consistently maintained in the canopy [2], [3]. Crown fires are extremely difficult to control due to their high rates of spread, intensity, flame lengths, spotting and fire severity, and are the major concern for fire managers and firefighters on safety, fire suppression and environmental developments [2], [4].

Given the inherent risks associated with crown fires, some experimental studies that obtained direct information from fires have been focused on low-intensity fires [4], [5], whereas measurements of extreme fire behavior associated with crown fires are more limited [6]–[8]. A widely used alternative method to obtain information from wildfires is based on multitemporal indices derived from remote sensing, which capture the substantial spectral changes that fire causes by consuming vegetation, destroying leaf chlorophyll, exposing soil and charring stems [9]. One of the most common variables measured by remote sensing is fire severity, defined as the degree of fire-induced environmental change on vegetation immediately after fire [10]. High values of fire severity are commonly related to crown fires and are important to validate fire risk maps, fire behavior models and management effectiveness [11], [12]. Moreover, the use of remote-sensing data allows the quantification of fire patterns over time and space, in particular the study of historical wildfires without available field data [13]. Landsat image data has been shown to classify accurately a large variety of landscapes, including the heterogeneous Mediterranean landscapes [14], [15] and this imagery is usually transformed into indices (such as Normalized Difference Vegetation Index (NDVI) [16] or the Normalized Burn Ratio (NBR) [17]) by rationing spectral bands to assess fire severity [18].

Fire severity depends on the combination of physical variables, weather and fuels. Concerning physical variables, in some studies topography has been shown to affect the pattern of fire severities [19], [20], whereas in other studies this effect is not clear and coincident [21], [22]. Weather variation is strongly related to fire severity. Specifically, low relative humidity, strong surface wind, unstable air and drought are described as the four critical weather elements of extreme fire behavior [6]. Regarding fuels, fuel moisture and forest structure are essential in determining the extreme fire behavior associated with crown fires [23]. But the way that fuels are related to fire severity is not obvious, as some authors suggest a clear relationship between both, even under drought conditions [13], [24], whereas in other studies, especially under infrequent high and mixed-severity fire regimes, the role of fuels is reduced [25], [26].

The European Project “Fire Paradox”[27] analyzed the spread of fire in historical wildfires and showed that there were similar spread schemes dominated by common factors (e.g. wind direction and speed). Depending on the spread scheme and the dominant spread factor, three fire types were defined: convection or plume-dominated fires, wind-driven fires and topographic fires [28], [29]. Firstly, convection or plume-dominated fires are characterized by the accumulation of high quantity of available fuels and atmospheric instability. This fire type has such a high intensity and extreme behavior that produces its own fire environment and generates massive spotting. Secondly, wind-driven fires follow the speed and direction of strong winds when the meteorological window that produces the fire conditions is maintained, with the same intensity and velocity during day and night. In both of them, small changes in the landscape have little influence in the direction and behavior of these fire types, especially under extreme meteorological conditions. In contrast, topographic fires are dominated by local winds caused by slope and differences in solar heating of the earth surface (i.e. sea breeze, land breeze, valley and slope winds). The direction of this fire type changes with topography (e.g. hydrographic basins, main valley), and it has high intensity during the day and low intensity at night [28], [29]. In the latter fire type, wildfire is more sensitive to small changes, thus little variations of topographical wind, slope or aspect have higher influence on fire behavior.

The combination of two or three fire types in the same wildfire might be common in North America, Canada and Australia, since fire usually burns during many days or months and involves large areas of the landscape. Nevertheless, the majority of wildfires in Europe burn for 48 hours or less, thus fire has fewer opportunities to flip from one fire type to another. Moreover, the characterization of these three fire types allows the identification of the operational opportunities for the suppression systems [28], [29]. Finally, these fire types are linked to meteorological conditions and topographical areas where they usually burn; thus, it is possible to know the risk of having one or another in the landscape depending on the meteorological forecast.

The number of severe wildfires and their recurrence have increased during recent years in the Mediterranean Basin, leading to an increase in wildfires characterized by crown fire and extreme fire behavior [30], [31]. Aleppo pine (*Pinus halepensis* Mill.) forests have been those most affected by high severity crown fires [31]. *Pinus halepensis* is one the most abundant conifers in the Mediterranean Basin, mostly at low elevations [32]. It is a serotinuos and not self-pruning species, with high vertical and horizontal continuity, which constitutes a highly flammable material that amplifies fire intensity from low-intensity surface fires to high-intensity crown fires [32], [33]. The present study is based on 15 historical wildfires of *P. halepensis* that occurred in Catalonia (NE Spain) in the period 2000–2007. The studied fires corresponded to the three fire types described above (i.e., topographic, convective and wind-driven fires) and included a wide range of fuel, weather and physical variables. The objectives of the study are: (1) to determine the variables that influence extreme fire severity among fires (considering the 15 wildfires together), and (2) to ascertain which variables affect extreme fire severity within each fire (15 wildfires of the three different fire types). To achieve these aims we have integrated the information available from firefighter reports and from different data bases generating new variables, some of which had never previously been considered.

## Materials and Methods

### Study area

**Figure 1:**
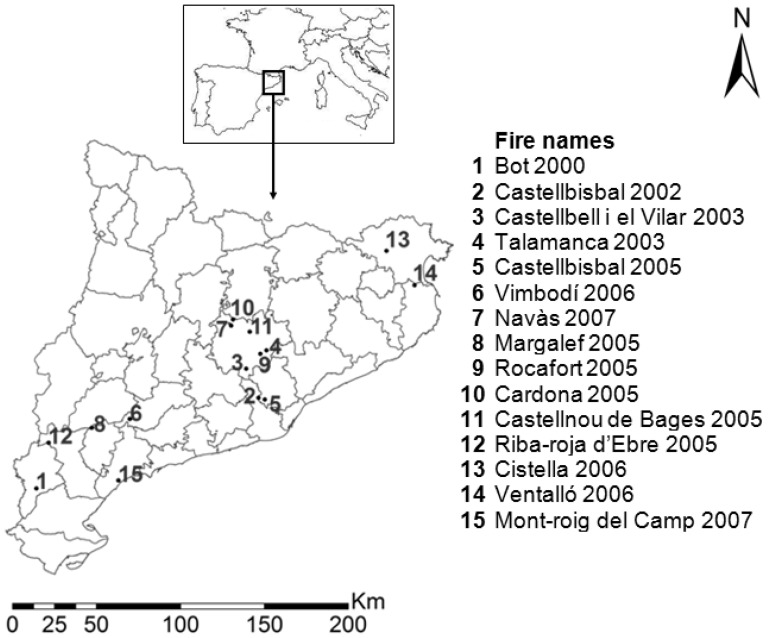
Names and location of the fires studied. Black points indicate the center of the fire and inner lines indicate regions.

The 15 fires studied were located in Catalonia, Northeastern Spain, between 40°57′ and 42°17′ latitude North and 0°22′ and 3°04′ longitude East (Figure 1). The climate is Mediterranean, characterized by mild winters and hot and dry summers, although Catalonia encompasses a large climatic gradient [34], [35]. Mean annual temperature ranged from 11.1 to 16.3°C, mean temperature of the hottest season (summer) from 19.3 to 24.1°C and mean temperature of the coldest season (winter) from 4.2 to 9.6°C. Mean annual precipitation ranged from 341 to 819 mm and mean precipitation of the driest season (summer) from 61 to 200 mm (Digital Climatic Atlas of Catalonia; [36], [37]).

The analyzed fires occurred between 2000 and 2007 and covered the three fire types according to the fire spread pattern: topographic, convection-dominated and wind-driven wildfires (Table S1). The selection of fires was determined by four conditions: (i) availability of cloud-free Landsat imagery before and after the fire, (ii) minimum forest cover within the fire of 30%, (iii) homogeneity in the fire type; and (iv) availability of information about fire behavior and fire effects from firefighter reports.

### Computing and mapping fire severity

Imagery used was obtained from Landsat Thematic Mapper (TM) and Enhanced Thematic Mapper Plus (ETM+), which was geometrically corrected following the Palà and Pons method [38], resulting in 20 m pixel size. Afterwards, a radiometric correction was applied to convert digital numbers (DN) to reflectance values, using a digital elevation model and parameters of exoatmospheric solar irradiance, atmospheric optical depth and sensor calibration [39]. The time between pre- and post-fire imagery was as short as possible (or near-anniversary date, [40]) in order to avoid phenologic changes of vegetation (Table S1).

We used the differenced Normalized Burn Ratio (dNBR) [41], [42], calculated from the Normalized Burn Ratio (NBR) for each image using bands 4 and 7, as shown in equation 1. The delta NBR (dNBR) was computed using the pre-fire minus the post-fire value of NBR (NBR_pre_ and NBR_post_ in equation 2). The GIS-software used was *MiraMon 7.0* [43].
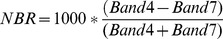
(1)

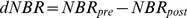
(2)


In our study, we used field data from plots sampled in the Ventalló wildfire (fire number 14 on Figure 1, [12]) to obtain the severity thresholds. In this wildfire, we considered three severity levels within individual trees: (1) green trees, with at least 20% green crown; (2) scorched trees, which had less than 20% green crown (although most of them were completely scorched); and (3) charred trees, which were skeletons mainly consumed without fine materials on the tree or on the ground [12]. Afterwards, three categories of severity at the plot level (20 m diameter) were defined: (1) green plots/low severity (at least 50% of green trees); (2) scorched plots/moderate severity (at least 50% of scorched trees and not more than 25% of green trees); and (3) charred plots/extreme severity (almost 100% of charred trees) [12]. We calculated the average dNBR value of a 3×3 pixel window centered in the plots of this fire and we obtained a data base containing the dNBR values and their corresponding severity level, thus defining ranges of dNBR and their number of charred and green-scorched plots (we grouped low and moderate severity). Afterwards, we defined the possible severity thresholds and we calculated the percentage of correct classification in every threshold (the percentage of correctly defined charred or green-scorched plots using this threshold). The severity thresholds were defined in order to maximize the correct classification of extreme severity. The best correct classification defined was 81% of charred plots and 59% of green-scorched plots. To obtain the threshold between unburned and burned forest pixels, we applied the same procedure to a selection of pixels from outside the fire. We also defined a burned threshold to shrub lands and crops to create a continuous map. We applied the thresholds defined in the Ventalló fire to the other fires and, together with using information from photographs obtained during and after the wildfires, firefighter reports and personal attendance to the fire events, we defined the final threshold limits by increasing the original threshold computed for Ventalló (Figure S1) by 5%.

From the information generated for the different fires we estimated the following severity variables in the two levels according to the two objectives: (i) in the first level, among fires (considering the 15 wildfires together), the variable was the percentage of forest pixels burned with extreme severity in the fire; (ii) in the second level, within fires, the variable was fire severity analyzed for each wildfire separately, which was categorical with two levels: not extreme severity (including both scorched and green severity) and extreme severity (charred severity).

### Fuels, topographic and fire behavior variables

In this section we reversed the order of the levels of variables, since many of the variables used in the first level (among fires) were calculated as average values of the variables used for the second level (within fires). We divided the independent variables considered at the two levels in three groups (Table 1).

**Table 1:** **I**ndependent variables of the three groups considered at the two levels: fuel distribution, topography and fire behavior, analyzed within and among fires.

Group of variables	Within fires	Among fires
**Fuel distribution**	Crown biomass (tons/ha)	Mean crown biomass (tons/ha)
	Water Availability Index (WAI)	Area with crown biomass >15 tons/ha (%)
	Drought Code	Climate type
		Mean Water Availability Index
		Mean Drought Code
**Topography**	Slope (°)	Mean slope (°)
	Aspect (south/north)	Area with slope higher than 20° (%)
	Elevation (m)	Area with southern aspect (%)
		Mean elevation (m)
**Fire behavior**	Type of slope (upslope/downslope)	Fire type (topographic, convective or wind-driven)
	Alignment (full alignment/out of alignment)	Temperature >30°C (yes/no)
		Relative humidity <30% (yes/no)
		Wind speed >30 km/h (yes/no)
		Urban interface within the fire (yes/no)
		Relative humidity recovered (yes/no)
		Area with upslope (%)
		Area with full alignment (%)

Units (for continuous variables) or levels (for categorical variables) are shown in brackets.

#### (1) Fuel distribution variables

Fuels are a key factor determining fire severity [44], [45]. This group includes variables related to fuels that can directly influence fire severity, such as crown biomass, [17] or indirectly through climatic characteristics that determine fuel quantity and distribution [33].

The variables in the within fires level are:

- Crown biomass (tons/ha), containing branches and leaves without the trunk. We used the crown biomass data of *P. halepensis* plots from the third National Forest Inventory of Spain [46]. As inventory data was not enough to cover the entire burned area, we tested the relationship between crown biomass and different spectral bands and combinations of bands that had been shown to assess better crown biomass [17] (Table S2). We selected the index showing the highest R^2^: MID57 (band 5 + band 7); R^2^ = 0.400, p<0.001, B_0_ = 42.177 and B_1_ = −0.061 (exponential regression), after the elimination of the outliers with standardized residual higher than 2 (Figure S2 and Table S3). We applied this relationship to the MID57 values of the studied fires to obtain the crown biomass value of each pixel.
- Annual Water Availability Index (WAI), obtained from the Digital Climatic Atlas of Catalonia [36], [37] and following the expression
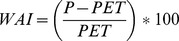
where *P* is rainfall (mm yr^−1^) and *PET* is potential evapotranspiration (mm yr^−1^). Negative values corresponded to dry sites and positive values to wet sites.
- Drought Code, a component of the Fire Weather Index that was calculated for each pixel following the methods of Van Wagner and Picket [47]. The required daily meteorological data of ten years before the date of ignition was previously corrected using elevation ranges.

The variables in the among fires level are:

- Percentage of forest area, determined from the Land Cover Map of Catalonia [48].
- Mean crown biomass, determined as the average value of crown biomass from all forested pixels within the burned area.
- Percentage of area with crown biomass >15 tons/ha, as crown biomass affects fire severity especially in dense forests with crown biomass over 15 tons/ha [17], determined from crown biomass at the pixel level.
- Climate type, according to the Thornthwaite moisture index (1948) classification [49]. We considered two main climates in the study area: semi-arid and not semi-arid (including dry-subhumid and humid).
- Mean WAI, the mean WAI of the total number of pixels of the fire.
- Drought code, developed by the Forest Fire Prevention Service of Catalonia following Van Wagner and Pickett [47] using the available data of all the years before the fire from the meteorological station closest to each fire.

#### (2) Topographic variables

From a Digital Elevation Model we selected topographic variables that are known to influence fire severity in many previous studies [16], [20], such as slope, aspect and elevation [19], [29]. Particularly, in 20° slopes head fire spread rates increase four times compared to flat terrain [50].

The variables in the within fires level are:

- Slope, in degrees.
- Aspect: south (90–270°) and north (270–90°).
- Elevation, in m.

The variables in the among fires level are:

- Mean slope (°), the mean slope of the total number of pixels of the fire.
- Percentage of area with slope higher than 20°.
- Percentage of area with southern aspect (90–270°).
- Mean elevation (m), the mean elevation of the total number of pixels of the fire.

#### (3) Fire behavior variables

We included variables related to fire risk and potential fire behavior: type of slope, alignment of factors, fire type, meteorological variables and presence of wildland-urban interface. The basic fire spread factors included were wind and slope, and their coincidence in favor of or against fire led to the concept of alignment of forces [29]. We also considered the basic rule of 30-30-30, which means that relative humidity under 30%, surface wind speed higher than 30 km/h and temperatures higher than 30°C facilitate the increase of fire spread [51].

The variables in the within fires level are:

- Type of slope. We defined two categories depending on slope (upslope or downslope) and wind effect (following wind or low wind effect). We determined the main wind direction of the fire from firefighter reports. Afterwards, the type of slope was defined from the closest topographic wind conditions, that is, from slope and aspect. Thus, the two categories defined were (i) upslope, when wind direction was following upslope, then the aspect of the slope was opposed to wind direction (at 135° of range with the wind direction as a center); and (ii) downslope, when there was leeward slope and the aspect was the same as wind direction.
- Alignment of factors: combination of type of slope (upslope or downslope) and slope >6° (more than a gentle slope), which was considered the minimum for fire alignment, resulting in two categories: (i) full alignment (upslope with wind following and slope>6°) and (ii) out of alignment (all the other combinations).

The variables in the among fires level are:

- Fire type, with three categories: (i) topographic fire, (ii) convection-dominated fire and (iii) wind-driven fire, according to the classification of the European project “Fire Paradox” [27] and obtained from the firefighter reports and personal attendance during the fires.
- Temperature >30°C during the fire, with two categories: (i) temperature over 30°C (yes) and (ii) temperature not over 30°C during the fire (no), obtained from the firefighter reports.
- Relative humidity <30% during the fire, with two categories: (i) relative humidity over 30% (yes) and (ii) relative humidity not over 30% during the fire (no), obtained from the firefighter reports.
- Wind speed >30 km/h during the fire, with two categories: (i) wind speed over 30 km/h (yes) and (ii) wind speed not over 30 km/h during the fire (no), obtained from the firefighter reports.
- Relative humidity recovered (higher than 60% the last night before the wildfire), considering two categories: (i) relative humidity recovered (yes) and (ii) relative humidity not recovered (no) (Official Firefighter reports from the wildfires analyzed).
- Presence of Wildland-urban interface. From the Firefighter reports we identified the fires within wildland-urban interface, i.e. those fires in which more resources were addressed to protecting property and people than to containing the wildfire, thus affecting their behavior and severity [29]. This variable had two categories: (i) fire in wildland-urban interface (yes) and (ii) fire not in wildland-urban interface (no).
- Type of slope: % of pixels with upslope and following wind within the burned area.
- Alignment of factors: % of pixels with full alignment (upslope and slope>6°).

### Data analysis

According to the objectives of the study, there were two levels of data analysis: among fires and within fires. The software used was *SPSS 17.0* (SPSS Inc., Chicago, IL, USA) and *R* (version 2.13.2, [52]).

At the among fires level, and given the high number of independent variables in relation to the number of cases, we carried out a multifactorial analysis to reduce the number of independent variables. For that reason, the independent variables were grouped in three groups in relation to fuel distribution, topography and fire behavior. We carried out separate Principal Coordinate Analyses (PCoA) to fuel distribution and fire behavior variables, and a Principal Component Analysis (PCA) to topographic variables. The results obtained (Figures S3, S4, S5) allowed us to select the variables explaining the highest variability in the first axes of the analysis of each of the three groups. We finally selected climate type and mean crown biomass from the fuel distribution group, alignment, urban interface and wind speed >30 km/h from the fire behavior group, and mean slope from the topography group. We carried out a General Linear Model (GLM) to evaluate the effect of these variables, together with fire type, on the proportion of the wildfire surface burned with highest severity. As this variable did not have a normal distribution, we transformed it by the arcsine of the square root to reach normality. Significance was assessed at p<0.05.

At the within fires level, as the number of pixels within the burned area of each fire was extremely high, we randomly selected 500 pixels (or all when there were fewer than 500 pixels in any level) of each of the two levels (not extreme severity/extreme severity) of the dependent variable. Previous correlation analysis among the explanatory variables allowed us to eliminate one of each pair of variables that were highly correlated (i.e., Pearson r≥0.9). With this procedure we excluded from the analyses Drought Code, elevation and type of slope (correlated with WAI and alignment of factors). Thus, the independent variables considered for these analyses were crown biomass, WAI, alignment, aspect and slope. For each fire separately, a Generalized Linear Model (GLZ) was carried out with binomial distribution and a logit link function. In each analysis, we included the main effects of the variables and the first order interactions. Stepwise model selection was applied starting from the saturated model and removing the least significant term, starting by the interactions until there was no further decrease in the Bayesian Information Criterion (BIC). We considered all models within 2 BIC units as equivalent in terms of fit.

## Results

### Variables affecting fire severity among fires

The General Linear Model carried out at the wildfire scale (R^2^ = 0.81; F = 10.4; p = 0.0013) showed that climate and slope significantly affected fire severity (Table 2). In the case of climate, fires that occurred in non- semi-arid climate showed more extreme fire severity than fires in semi-arid climate (0.71±0.04 and 0.21±0.07 parts per unit, respectively). Concerning slope, the relationship of this variable with the percent of the area burned with extreme severity was positive, indicating that fires on steeper slopes had more extreme severity than those on gentle slopes.

**Table 2:** Statistical results from the General Linear Model for the effects of climate, wildland-urban interface, crown biomass and slope on extreme severity.

Source of variation	SS	d. f.	MS	F	p
Climate	0.305	1	0.305	11.932	**0.006**
Wildland-urban interface	0.019	1	0.019	0.734	0.412
Crown biomass	0.008	1	0.008	0.305	0.593
Slope	0.140	1	0.140	5.461	**0.042**
Error	0.256	10	0.026		

SS, sum of squares; MS, mean square.

Significant results at p<0.05 are indicated in bold.

### Variables affecting fire severity within fires

**Figure 2:**
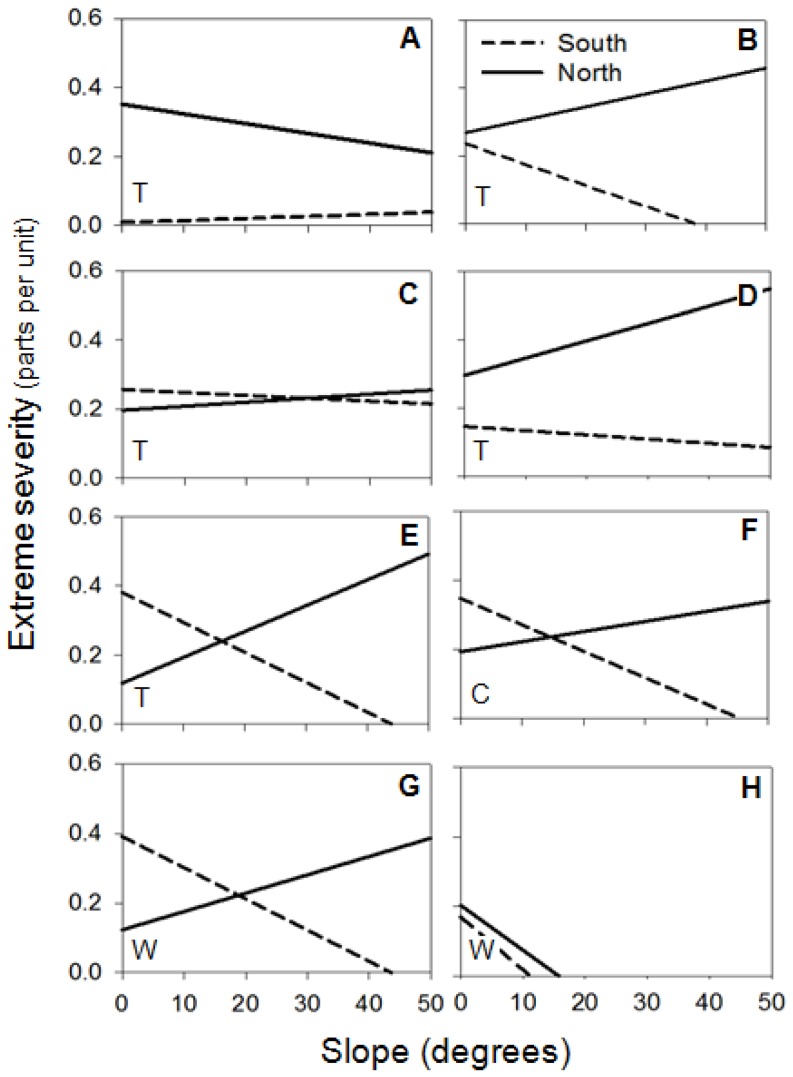
Significant interactions of Aspect (north or south) and Slope (degrees) on extreme fire severity (parts per unit). Letters in the top-right corner indicate the fire: (A) Bot 2000; (B) Castellbell i el Vilar 2003; (C) Castellbisbal 2005; (D) Vimbodí 2006; (E) Navàs 2007; (F) Castellnou de Bages 2005; (G) Cistella 2006; (H) Mont-roig del Camp 2007. Letters in the bottom-left corner indicate the fire type: (T) topographic fires; (C) convective fires; (W) wind-driven fires.

Table 3 summarizes the GLZ analyses carried out separately with the fifteen fires considered in this study. Regarding topographic variables, aspect was significant in 73% of fires (71% of topographic, 75% of convective and 75% of wind-driven fires) (Table 3), showing in all but one case more extreme severity in northern than in southern aspects. Slope was significant in 73% of fires, with more extreme severity on steeper than on gentle slopes in all fires except one (Table 3). The interaction between these two topographic variables was significant in 53% of fires, showing on steeper slopes more extreme severity in northern than in southern aspects in 88% of these fires, while on gentle slopes the degree of extreme severity was less different or even higher in southern than in northern aspects (Figure 2).

**Table 3:** Statistical results from the Generalized Linear Model for the main effects and interactions on extreme severity in each fire.

		TOPOGRAPHIC FIRES							CONVECTION-DOMINATED FIRES				WIND-DRIVEN FIRES			
		Bot 2000	Castellbisbal 2002	Castellbell i el Vilar 2003	Talamanca 2003	Castellbisbal 2005	Vimbodí 2006	Navàs 2007	Margalef 2005	Rocafort 2005	Cardona 2005	Castellnou de Bages 2005	Riba-roja d'Ebre 2005	Cistella 2006	Ventalló 2006	Mont-roig del Camp 2007
Aspect (ASP)	B	−39.00		−25.00	−0.95	−18.00		−7.40	−3.70		−1.80	−4.40	−1.09	1.50		−0.91
	Wald	5.5		13.8	13.6	9.0		7	20.0		6.9	14.0	8.5	18.0		7.0
	sig	**0.019**		**0.000**	**0.000**	**0.003**		**0.008**	**0.000**		**0.008**	**0.000**	**0.030**	**0.000**		**0.008**
Slope (SLP)	B	0.13		1.02	0.30	1.30			0.08	0.34	0.68	0.61	1.10		1.30	−22.40
	Wald	30.0		4.9	43.0	15.0			41.0	10.0	50.0	-	7.4		10.0	4.1
	sig	**0.000**		**0.026**	**0.000**	**0.000**			**0.000**	**0.002**	**0.000**	**0.003**	**0.006**		**0.001**	**0.042**
Crown Biomass (BIO)	B	1.70		−9.10	−0.92	0.24	1.90	3.00	−3.10	−3.10	0.25	0.32	−6.90	0.37	2.20	0.32
	Wald	137.0		68.0	5.8	141.0	7.5	60.0	5.8	26.7	35.0	80.0	13.0	182.0	21.0	6.7
	sig	**0.000**		**0.000**	**0.015**	**0.000**	**0.006**	**0.000**	**0.015**	**0.000**	**0.000**	**0.000**	**0.000**	**0.000**	**0.000**	**0.010**
WAI	B	0.50		2.30	0.46		−0.84	−0.69	0.52	1.12	−0.22	−0.22			−0.88	3.10
	Wald	60.0		63.0	11.7		8.8	21.1	6.3	47.0	25.0	5.5			11.0	10.1
	sig	**0.000**		**0.000**	**0.001**		**0.003**	**0.000**	**0.012**	**0.000**	**0.000**	**0.019**			**0.001**	**0.001**
Alignment (ALI)	B	−5.50	−1.70	−0.85	−3.30	24.50	14.00	−5.55	1.80				2.00	23.00		
	Wald	8.7	5.9	13.7	14.0	15.0	10.8	5.1	4.2				85.0	20.0		
	sig	**0.003**	**0.014**	**0.000**	**0.000**	**0.000**	**0.001**	**0.023**	**0.039**				**0.000**	**0.000**		
ASP x SLP	B	0.72		−0.11		−0.08	0.07	−0.12				−0.07		−0.86		−1.10
	Wald	5.9		23.0		17.4	4.3	28.0				6.9		9.0		18.0
	sig	**0.015**		**0.000**		**0.000**	**0.037**	**0.000**				**0.008**		**0.002**		**0.000**
ASP x BIO	B	3.70	−0.47						0.47		0.13	0.33				0.28
	Wald	4.8	4.1						19.0		9.0	22.0				4.0
	sig	**0.027**	**0.042**						**0.000**		**0.002**	**0.000**				**0.044**
BIO x WAI	B			−0.20	−0.04		0.04	0.06	−0.07	−0.08			−0.13		0.07	
	Wald			71.0	38.0		5.1	48.0	7.1	29.0			17.0		18.0	
	sig			**0.000**	**0.000**		**0.023**	**0.000**	**0.007**	**0.000**			**0.000**		**0.000**	
SLP x WAI	B			0.02		0.03					0.01	0.01	0.01		0.04	−0.48
	Wald			4.6		13.6					35.0	8.0	5.0		8.0	4.2
	sig			**0.031**		**0.000**					**0.000**	**0.005**	**0.018**		**0.004**	**0.039**
ALI x WAI	B					0.67	0.34	−0.14			0.09			0.81		
	Wald					17.0	12.0	6.1			4.9			13.0		
	sig					**0.000**	**0.001**	**0.013**			**0.026**			**0.00**		
ASP x WAI	B			−0.59		−0.51		−0.20								−1.90
	Wald			15.0		10.0		10.0								6.6
	sig			**0.000**		**0.002**		**0.002**								**0.010**
ASP x ALI	B					1.00			−1.19							
	Wald					7.8			5.9							
	sig					**0.005**			**0.150**							
ALI x BIO	B				0.33						0.10			−0.27		
	Wald				21.0						4.0			17.0		
	sig				**0.000**						**0.044**			**0.000**		
ALI x SLP	B						0.09		−0.13			0.10				
	Wald						8.2		13.0			9.0				
	sig						**0.004**		**0.000**			**0.002**				
BIO x SLP	B		−0.05		−0.02						−0.01					
	Wald		9.8		38.0						36.0					
	sig		**0.002**		**0.000**						**0.000**					

B, Wald statistic and significance at p<0.05 are shown.

**Figure 3:**
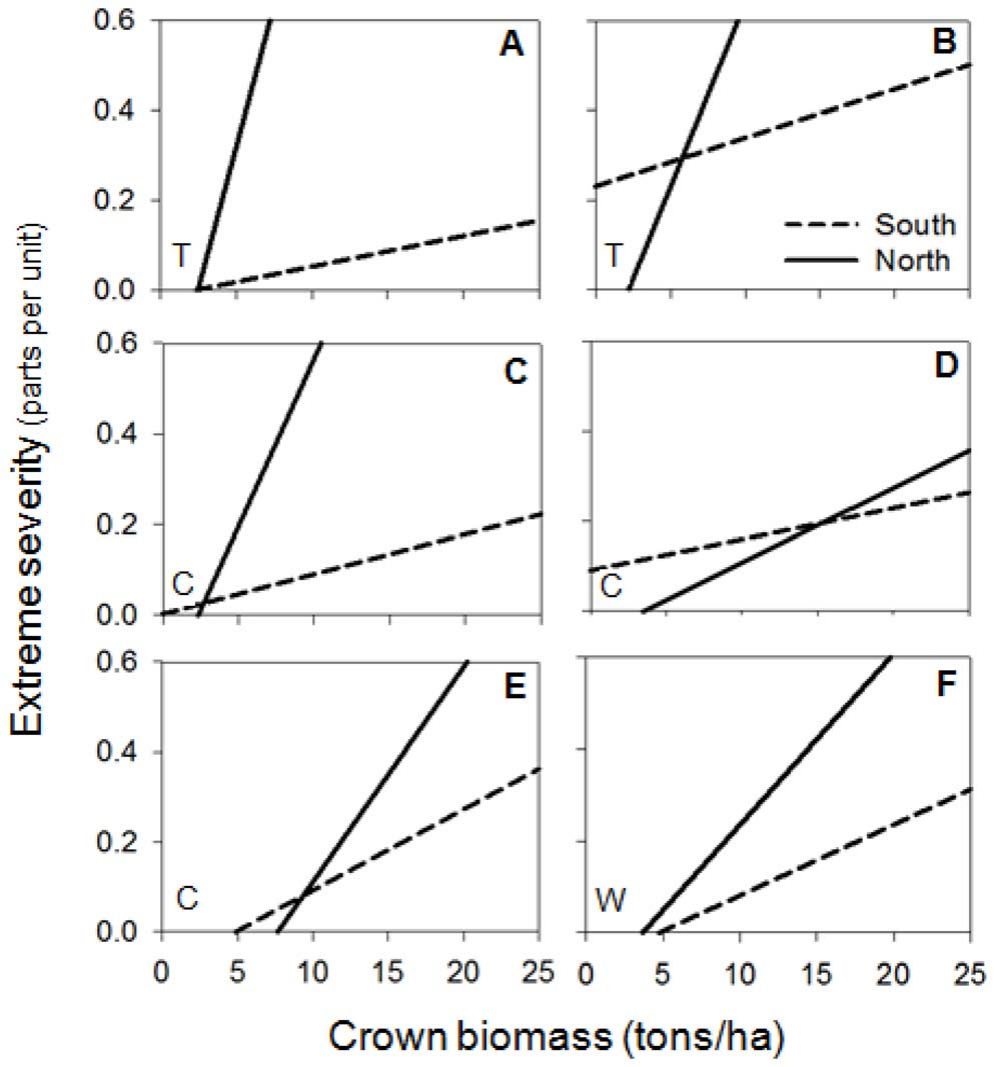
Significant interactions of Aspect (north or south) and Crown Biomass (tons/ha) on extreme fire severity (parts per unit). Letters in the top-right corner indicate the fire: (A) Bot 2000; (B) Castellbisbal 2002; (C) Margalef 2005; (D) Cardona 2005; (E) Castellnou de Bages; (F) Mont-roig del Camp 2007. Letters in the bottom-left corner indicate the fire type: (T) topographic fires; (C) convective fires; (W) wind-driven fires.

**Figure 4:**
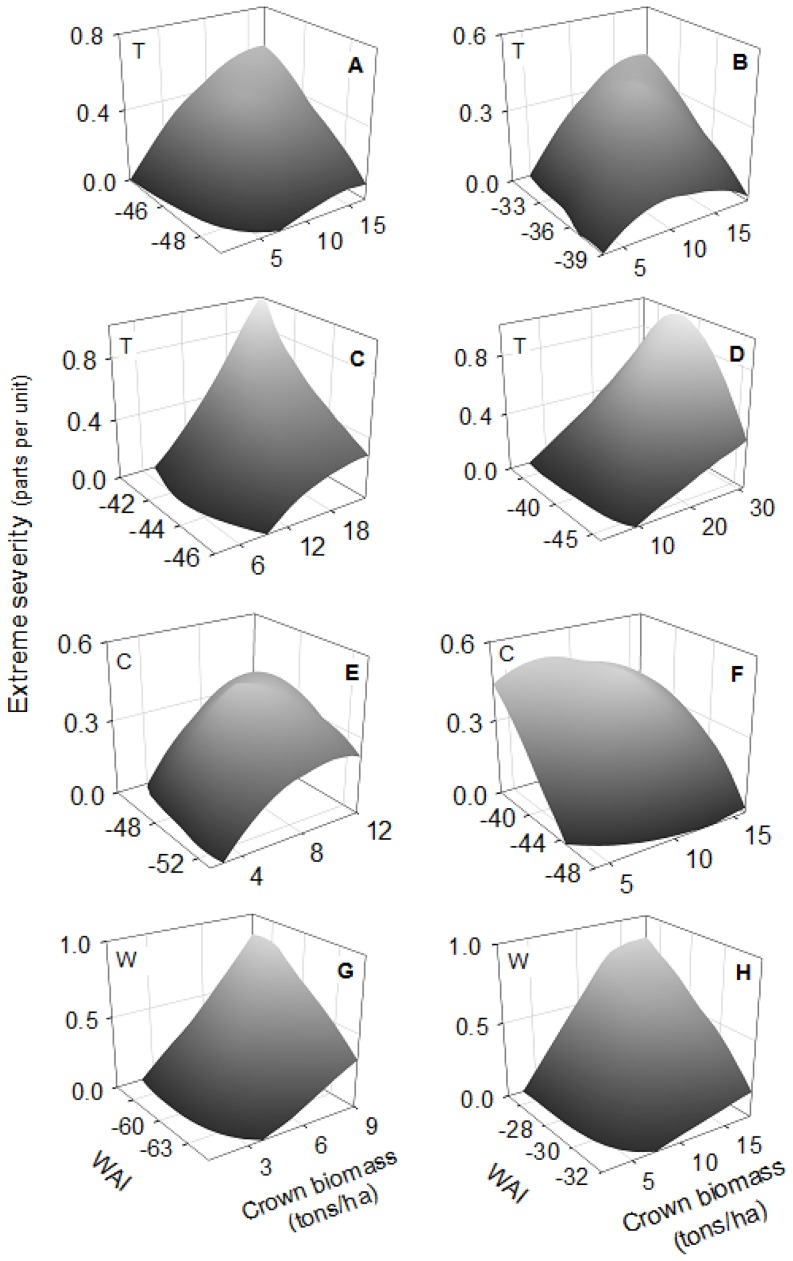
Significant interactions of Crown Biomass (tons/ha) and WAI on extreme fire severity (parts per unit). Letters in the top-right corner indicate the fire: (A) Castellbell i el Vilar 2003; (B) Talamanca 2003; (C) Vimbodí 2006; (D) Navàs 2007; (E) Margalef 2005; (F) Rocafort 2005; (G) Riba-roja d'Ebre 2005; (H) Ventalló 2006. Letters in the top-left corner indicate the fire type: (T) topographic fires; (C) convective fires; (W) wind-driven fires.

**Figure 5:**
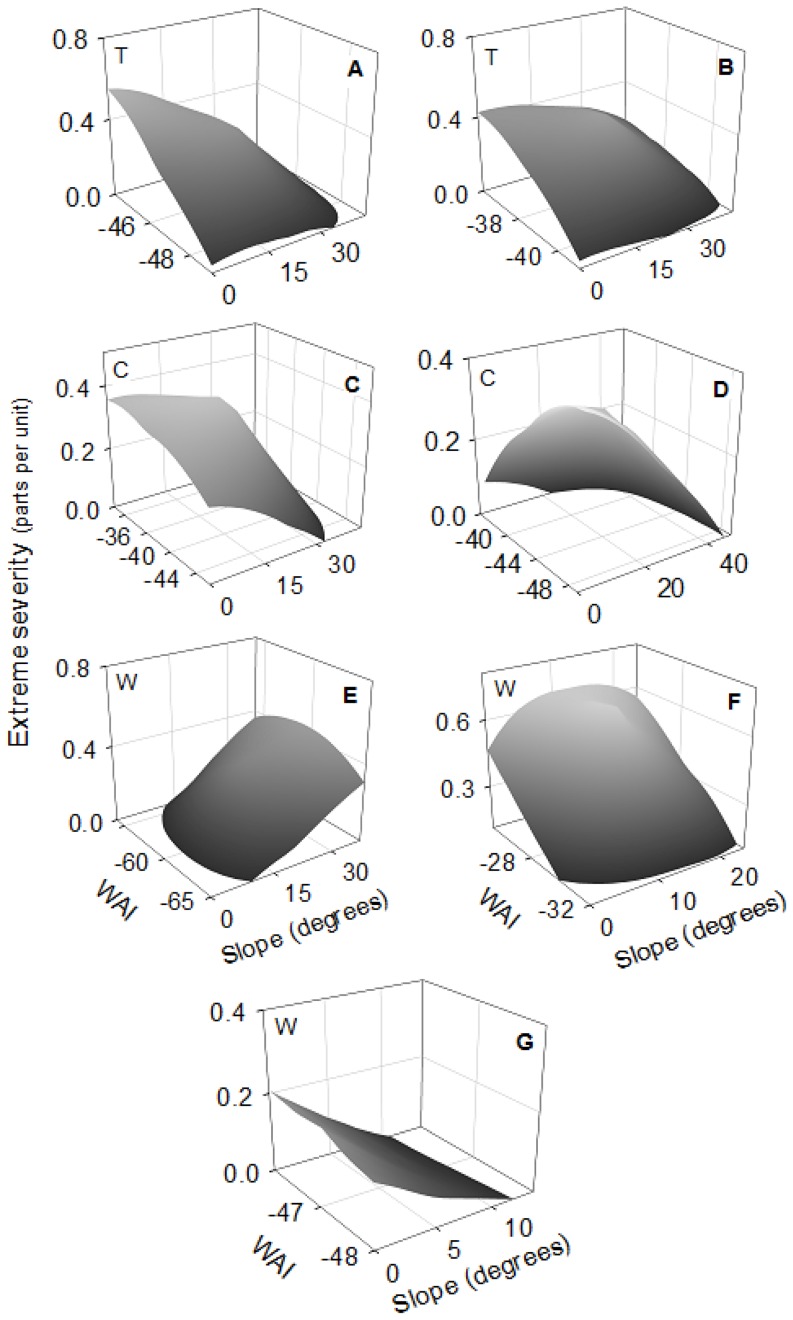
Significant interactions of Slope (°) and WAI on extreme fire severity (parts per unit). Letters in the top-right corner indicate the fire: (A) Castellbell i el Vilar 2003; (B) Castellbisbal 2005; (C) Cardona 2005; (D) Castellnou de Bages 2005; (E) Riba-roja d'Ebre 2005; (F) Ventalló 2006; (G) Mont-roig del Camp 2007. Letters in the top-left corner indicate the fire type: (T) topographic fires; (C) convective fires; (W) wind-driven fires.

Concerning fuels, crown biomass was significant in 93% of fires (86% of topographic and 100% of convective and wind-driven fires). The positive relationship was dominant, indicating more extreme severity at higher crown biomass, especially in topographic and wind-driven fires (Table 3). WAI was significant in 73% of fires (71% of topographic, 100% of convective and 50% of wind-driven), but the direction of the relationship was not homogeneous. The positive relationship predominated in topographic fires (60%), showing that at more WAI there was more extreme severity (Table 3). In convective and wind-driven fires that showed significant WAI, only half of the fires showed a positive relationship. The interaction between aspect and crown biomass was significant in 40% of the fires and it was only relevant in convection-dominated fires (75% of cases), indicating more extreme severity in northern than in southern aspects with high crown biomass, but small differences in extreme severity between aspects with lower crown biomass (Figure 3). The interaction between crown biomass and WAI was significant in 53% of fires (57% of topographic, 50% of convective and 50% of wind-driven) (Table 3). The pattern was similar in all fires except one, showing more extreme severity in higher crown biomass and higher WAI (Figure 4). The interaction between slope and WAI was significant in 47% of fires (28% of topographic, 50% of convective and 75% of wind-driven) (Table 3). But the direction of the relationship was not clear; although 57% of these fires had more extreme severity at low slope and high WAI, this increase was very slight (Figure 5). The remaining interactions were not considered, as the number of significant cases was lower than 33% and the relationship was not clear (Table 3).

Finally, alignment was significant in 67% of fires (100% of topographic, 25% of convective and 50% of wind-driven). The negative relationship was predominant in topographic fires (71%), showing more extreme severity when the fire was out of alignment. The relationship was positive in the two wind-driven fires that showed significant alignment (Table 3).

## Discussion

The spatial distribution of fire severity immediately after fire is a key question for validating fire risk maps, fire behavior models and fuel management effectiveness [11]. Previous works in the U.S.A. carried out with extensive databases using historical wildfires at national and regional scales have shown the utility of these studies to identify trends about size, frequency and percentage of high severity in fires [53], [54]. In our study, the innovative integration of different sources of information (digital cartography, firefighter reports, Landsat images) from historical wildfires across a range of climatic conditions, allowed us to determine patterns and variables affecting extreme fire severity at different levels, among and within fires.

### Variables affecting fire severity among fires

The analysis among fires shows that climate and slope are the main variables affecting the degree of extreme fire severity (Table 2). The linkage between climate and fire severity is widely known [26], [55]. Climate influences fire regime and forest growth through primary productivity, which is related to fuel accumulations that influence fire behavior and fire severity [45], [56], [57]. Previous studies have shown that the increase in wildfire frequency and fire severity was associated to dry climates, as drought decreases fuel moisture and, consequently, fuel flammability increases [58], [59]. But we found the opposite effect, indicating that fires in less arid climates had more extreme severity than those that occurred in more arid climates. In the Mediterranean area, plant growth is primarily constrained by water availability [60], [61], suggesting in our study that less arid climates had enough water for forest growth that facilitated crown fires and extreme fire severity. Contrarily, it is accepted that dry conditions reduce vegetation growth, thus leading to a lower accumulation of fuels in terms of quantity and continuity [62]. Specifically, the forest structure predominant in arid conditions is characterized by open forest with very low horizontal continuity of tree crowns , where fire is not likely to spread as an active crown fire, thus reducing extreme fire severity [33]. This was supported by Figure S6, where crown biomass was significantly lower in a semi-arid climate than in a less arid one. Furthermore, the percentage of forest was higher in the less arid climate, which also had more canopy closure (Figure S7). In forest structures characterized by high canopy closure, the risk of active crown fires is higher because there is an increase in vertical and horizontal continuity [3], [23], [63].

Although some studies have shown that, especially under extreme climatic conditions, climate exerts a dominant control on fire severity and fire behavior, the role of topographic features could also be important [64], [65]. Our study shows that slope is significantly higher in fires with more extreme severity. As slope increases, the distance and angle between the flame and fuels is shorter, facilitating the pre-heating of the fuel ahead of the fire front, and thus increasing fire spread [29]. Previous research by different authors has shown that fire severity increased with slope in the direction of fire progression [66]–[69], while the work of Lentile *et al*. [70] suggested that on steeper slopes the crowns of large trees were stacked up, thus facilitating fire spread from crown to crown and resulting in more extreme fire severity.

### Variables affecting fire severity within fires

In Catalonia, where this study has been carried out, there are areas dominated by different fire types, depending on fire spread pattern (topographic, convection, wind-driven) [29]. The variables affecting fire severity among fires were also significant in most of the fires within fires level, but there were more variables determining extreme fire severity at this particular level of analysis.

The topographical variables of aspect and slope are key variables in the majority of fires, showing that there is more extreme severity in northern aspects and on higher slopes. Some previous studies have shown that, in the Northern Hemisphere, southern aspects tend to burn with greater intensity and the resultant severity was higher than in other aspects, as southern aspects received more solar radiation and fuel moisture was lower [65], [71]. But in southern aspects growth limitations are also common and for this reason the effect of topographical variables is not always coincident [22], [72]. The results of our study indicate that extreme severity was more frequent in northern aspects (Table 3), where solar radiation was lower and fuels had less water limitation to growth, which could promote higher levels of tree density and vertical growth, as was shown in the Al Omary study [73]. This later pattern explains why northern aspects have higher crown biomass, promoting high fuel continuity and more extreme severity [19], [69], [73]. Slope showed the same tendency as in the previous among fires analysis, with more extreme severity on steeper slopes. The interaction between slope and aspect was also significant especially in topographic fires for a similar reason: the degree of extreme severity was highest on steeper slopes and northern aspects (Figure 2), as in this combination of variables fire spread and fuel continuity could be the highest. These findings were supported by the fuel results of the GLZ models, since there was more extreme severity at high values of crown biomass, especially in topographic and wind-driven fires (Table 3). Higher values of crown biomass affect fire behavior by increasing the area affected by crown fires and producing more extreme severity [32], [74].

Alignment of factors is a complex variable that cannot usually be estimated, as there is no information about variables such as wind direction at landscape scale. In our study, alignment of factors was significant in nearly 67% of wildfires, but the pattern was not homogeneous and higher alignment of factors implied higher extreme fire severity in only 50% of the cases. Although we expected that fire severity would increase with the combination of high upslope and wind direction, the opposite trend was observed for topographic fires and no trend was observed for convective and wind-driven fires. This is likely due to other factors that could modify the pattern of fire severity, such as forest structure, fuel moisture or the type of spread (head, flank or back fire), thus not all the areas aligned burned with the same severity. In topographic fires, we found the opposite trend because wind is not the most important factor explaining fire severity and behavior, as these fires are caused by complex relationships among fuel heating, slope and topographical winds [29].

Topographic fires showed a dominant pattern of more extreme severity at higher values of WAI (Table 3). This confirms the pattern mentioned among fires, where lower water limitations could lead to higher crown biomass and continuity that increase the proportion of extreme severity [12], [69]. In wind-driven and convective fires, this evidence was not clear probably because other variables were interacting at the same time. Wind-driven fires are characterized by high wind speed that could minimize the effect of WAI in determining extreme fire severity. In the same way, convective fires generate their own fire environment, thus reducing the effect of other local variables on spread [29]. Although WAI as a main variable was not significant in convective fires, in this type of fire there was more extreme severity in northern than in southern aspects with high crown biomass that can be related to water content (Figure 3). Convective fires are dominated by the accumulation of highly available fuel that is higher at northern aspects [25]. Moreover, even though there was more extreme severity at high crown biomass, this increase was much faster at higher values of WAI, especially in topographic fires (Figure 4). In less arid environments (where WAI is higher), flammability is lower and the quantity of heat necessary to start the ignition is higher [59]. Nevertheless, when fuels are available for burning under extreme meteorological conditions, they can burn as high intensity crown fires, thus involving more extreme severity [26], [75]. This pattern has recently been described in the Mediterranean area, where fires of high intensity and severity occur in humid forests that are not very prone to burn, such as some montane (sub-Mediterranean) areas of *Pinus nigra* and *Pinus sylvestris* [76], [77] and high mountain forests of *Pinus uncinata* in the Pyrenees ([78], Albert Alvarez, personal observation).

### Limitations and future implications

The analysis of historical wildfires is used to identify the main type of spread pattern in each area of a country and, thus, to know where it is more probable to have one of the three fire types. The probability to have a type of fire can be mapped and it is useful for firefighters to know the strategies, opportunities and critical points that they should use to control the fire before it starts. The use of the fire type concept is also useful to drive fuel management strategies through the definition of the Strategic Management Points (SMP), which are the key points where it is necessary to create infrastructures to limit the extent of large wildfires [29]. The use of remotely sensed data and their transferability to the dNBR index allowed us to determine the variables and patterns affecting fire severity using historical wildfires that could not be studied by other means. Our results suggest that this methodology could be applied to other fires in the Mediterranean area in a systematic way, but information from the “real” fire severity from some wildfires is needed, using direct data from firefighters or from post-fire plots in order to improve the accuracy in the definition of the severity thresholds and the validation of final fire severity maps. We also believe that the methodology used in this work could be improved by including additional information from forest structures. The use of Landsat time-series metrics, LIDAR or radar remote-sensing instruments (e.g., Pol-SAR) has recently been suggested as an effective way to provide information from aboveground biomass and forest structures [79], [80]. The combination of these data sources could improve the methodology in detecting specific areas such as the flanks of the fire runs that were probably not identified correctly.

Global climate change is increasing the number, severity and recurrence of forest fires, as well as the surface burned and the length of the fire risk season in the Mediterranean Basin [81]. Water scarcity and the expected increase of extreme droughts caused by climate warming will lead to an increase in high-intensity crown fires in areas that have not traditionally been subject to this type of fire ([78], Albert Alvarez, pers. obs.). Consequently, future research and management strategies to reduce future wildfires with crown fires and extreme fire severity should be focused not just on the most commonly burned areas but also on the less usually burned, since these new zones with high crown biomass accumulated in non-common drought areas will be available to burn as extreme severity wildfires.

## Supporting Information

Figure S1 **dNBR thresholds limits for forest (A), shrublands and fruit tree crops (B) and other crops (C).**
(TIF)

Figure S2 **Exponential regression between Crown Biomass (tons/ha) and MID57 index.**
(TIFF)

Figure S3 **Principal Coordinate Analysis (PCoA) of fuel distribution variables.**
(TIFF)

Figure S4 **Principal Coordinate Analysis (PCoA) of fire behavior variables.**
(TIFF)

Figure S5 **Principal Component Analysis (PCA) of topographic variables.**
(TIFF)

Figure S6 **Relation between climate and biomass (tons/ha).**
(TIFF)

Figure S7 **Relation between climate and forested area (%).**
(TIF)

Table S1 **Information about the fires analyzed.** Fire name, date of ignition, path/row, pre-fire data, post-fire data, sensor of the images, fire size, range of elevation, range of slope and fire type.
(PDF)

Table S2 **Bands related to Crown Biomass, regression type, B_0_(constant), B_1_(regression coefficient) and R square.**
(PDF)

Table S3 **Parameter estimates of the regression between Crown Biomass and MID57 index.**
(PDF)
