# Supplementary figures and images for: Extreme Fire Severity Patterns in Topographic, Convective and Wind-Driven Historical Wildfires of Mediterranean Pine Forests
Source: PLoS One. 2014 Jan 22;9(1):e85127. doi: 10.1371/journal.pone.0085127 (PMC3899010; doi:10.1371/journal.pone.0085127)

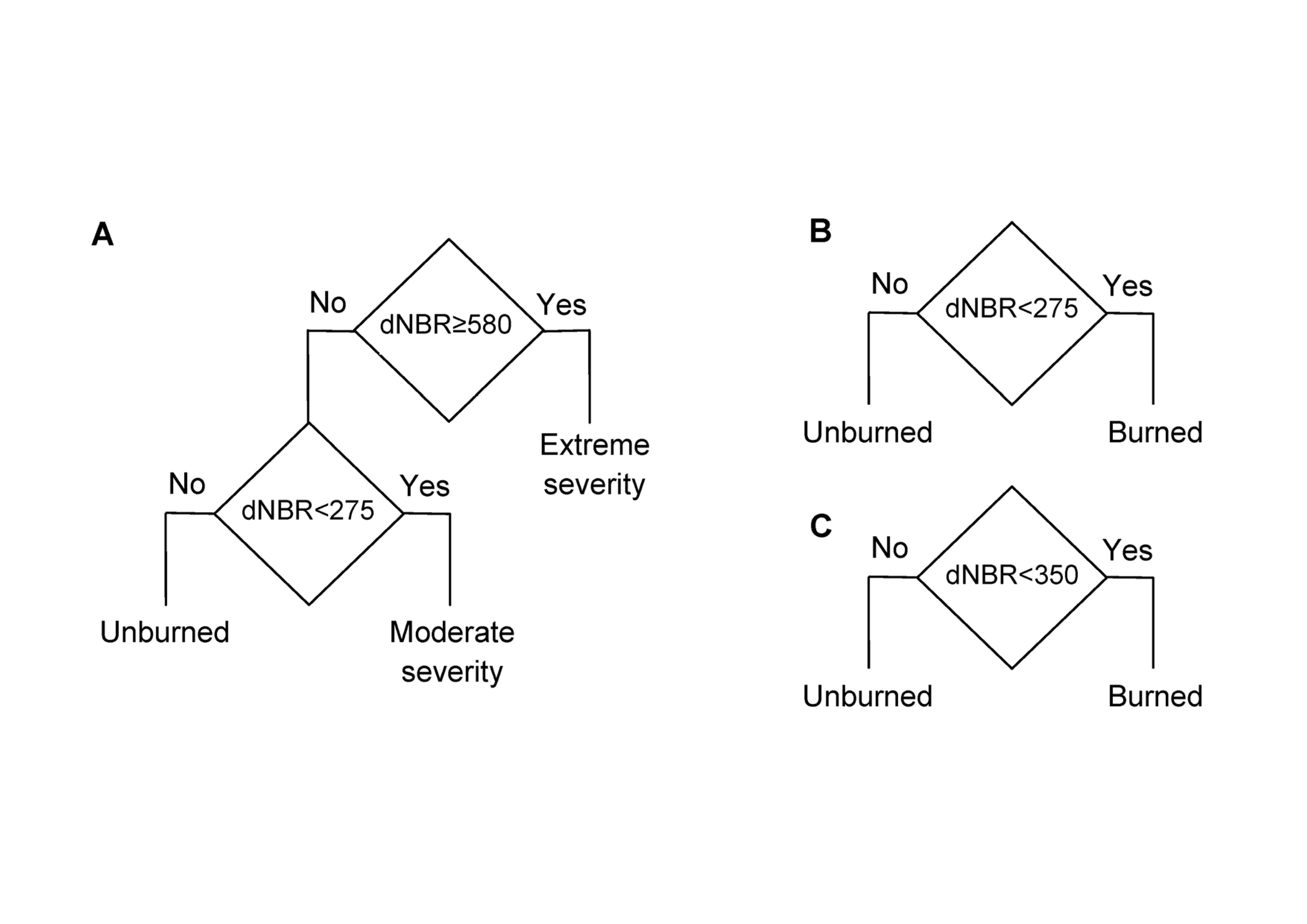

Supplement: Figure S1 — dNBR thresholds limits for forest (A), shrublands and fruit tree crops (B) and other crops (C). (TIF) [file pone.0085127.s001.tiff]

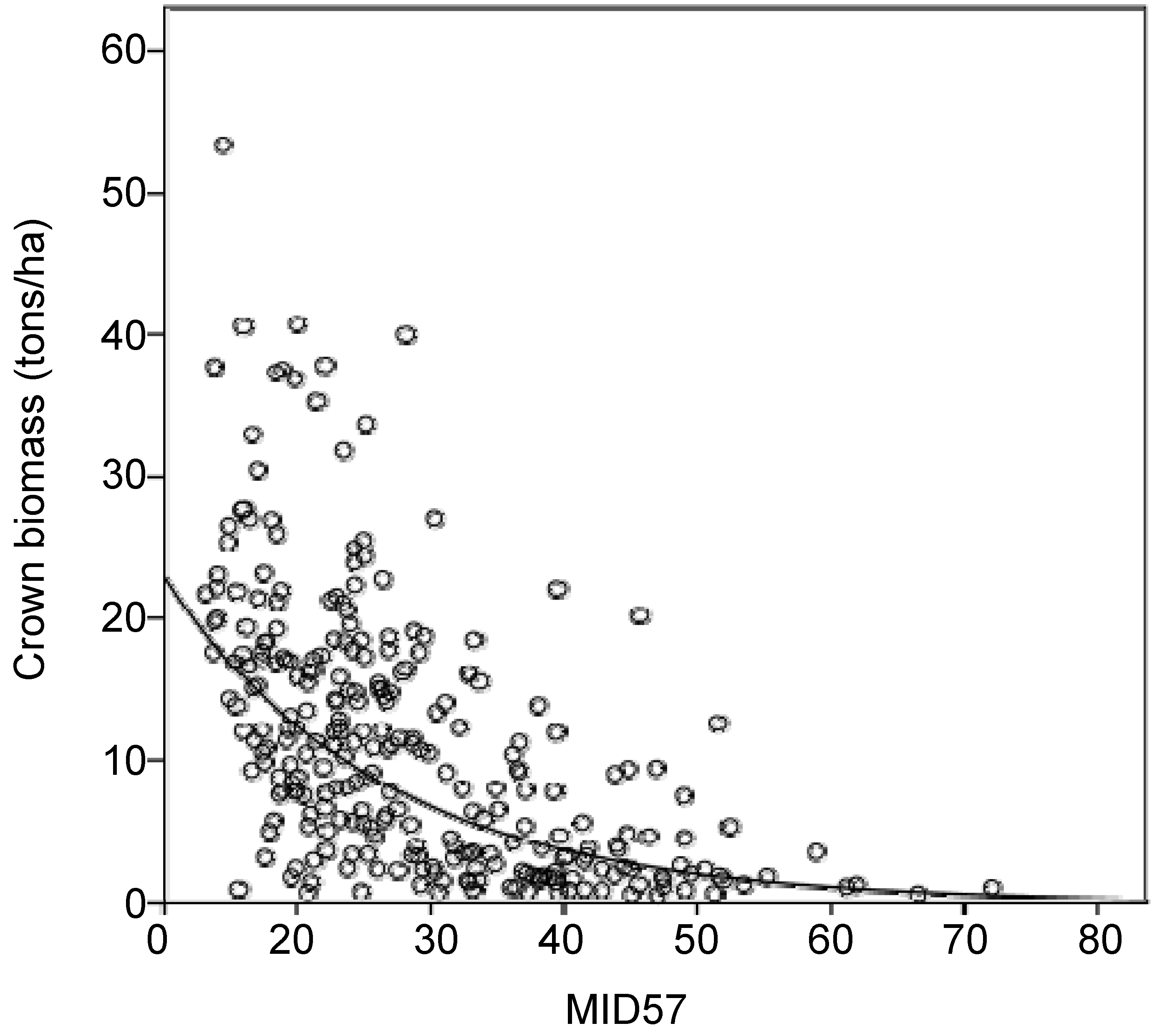

Supplement: Figure S2 — Exponential regression between Crown Biomass (tons/ha) and MID57 index. (TIFF) [file pone.0085127.s002.tiff]

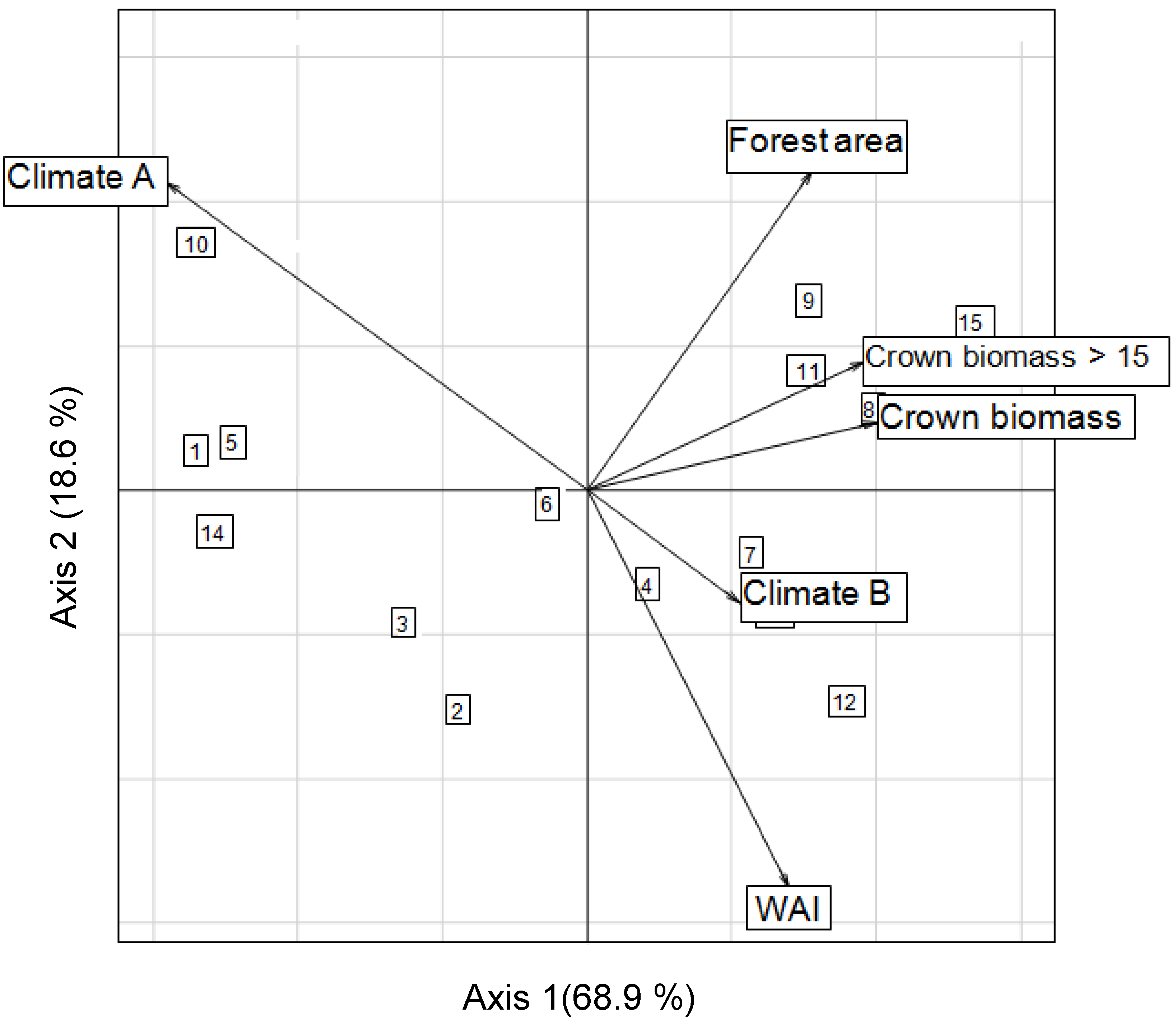

Supplement: Figure S3 — Principal Coordinate Analysis (PCoA) of fuel distribution variables. (TIFF) [file pone.0085127.s003.tiff]

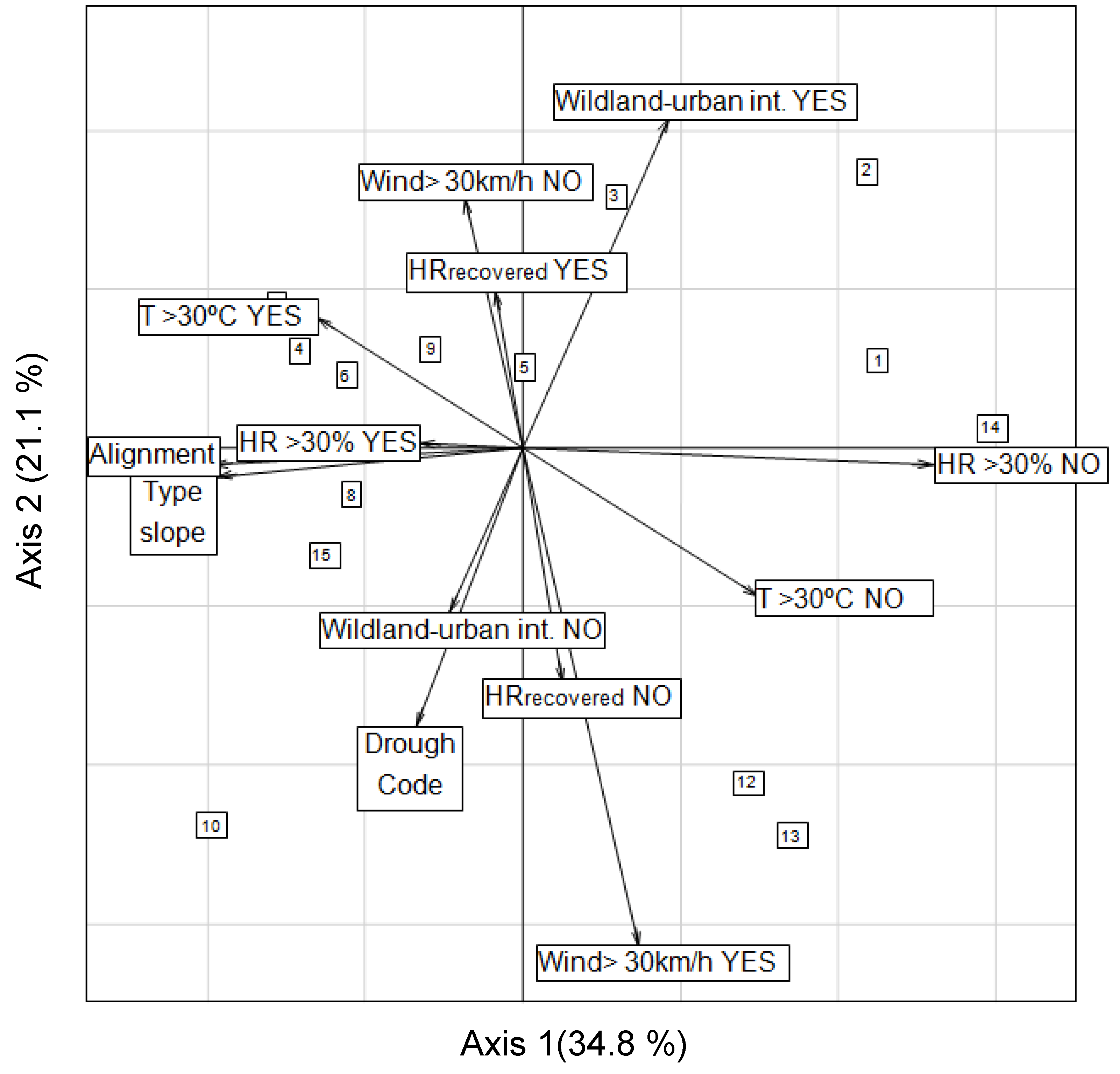

Supplement: Figure S4 — Principal Coordinate Analysis (PCoA) of fire behavior variables. (TIFF) [file pone.0085127.s004.tiff]

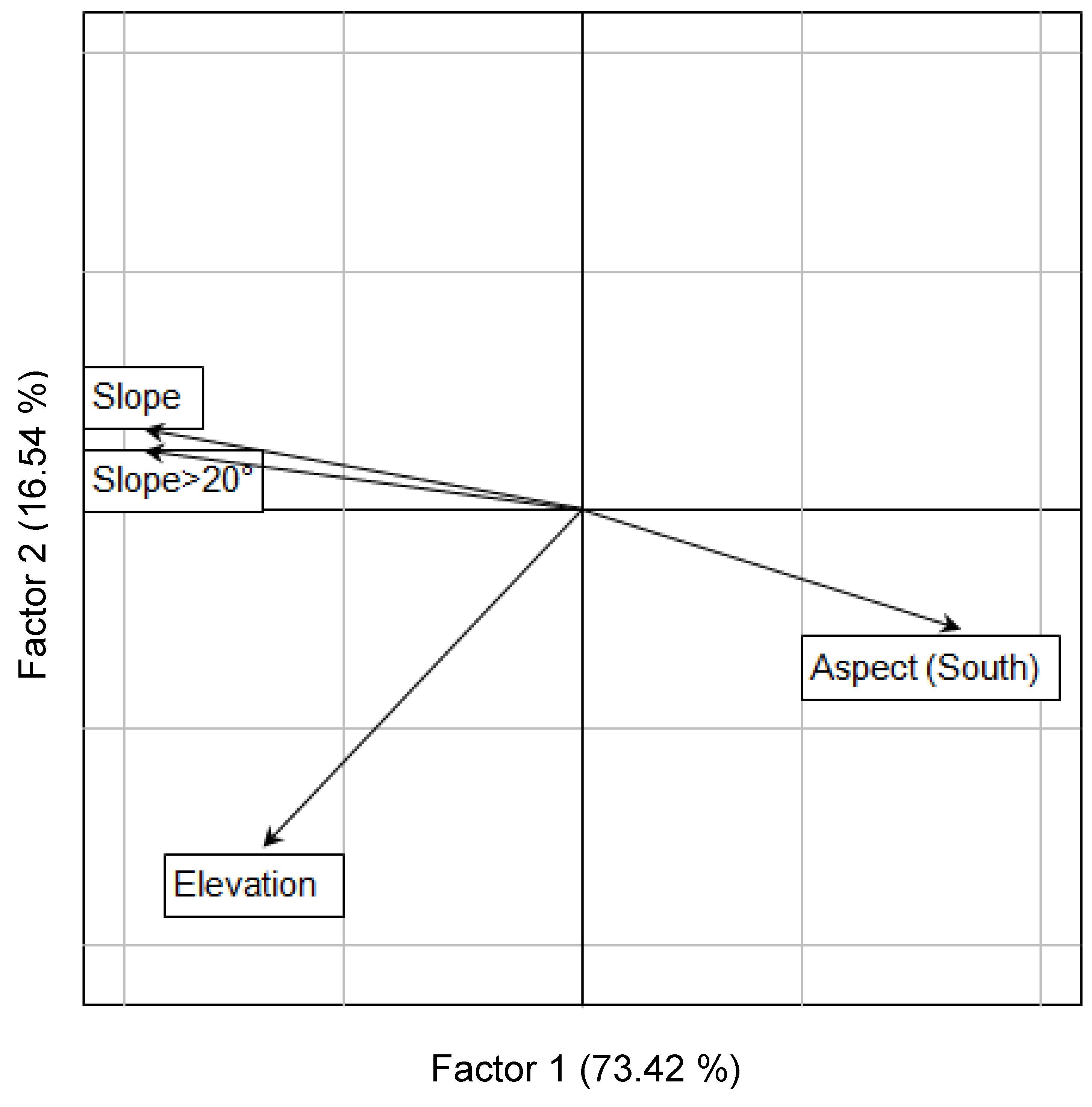

Supplement: Figure S5 — Principal Component Analysis (PCA) of topographic variables. (TIFF) [file pone.0085127.s005.tiff]

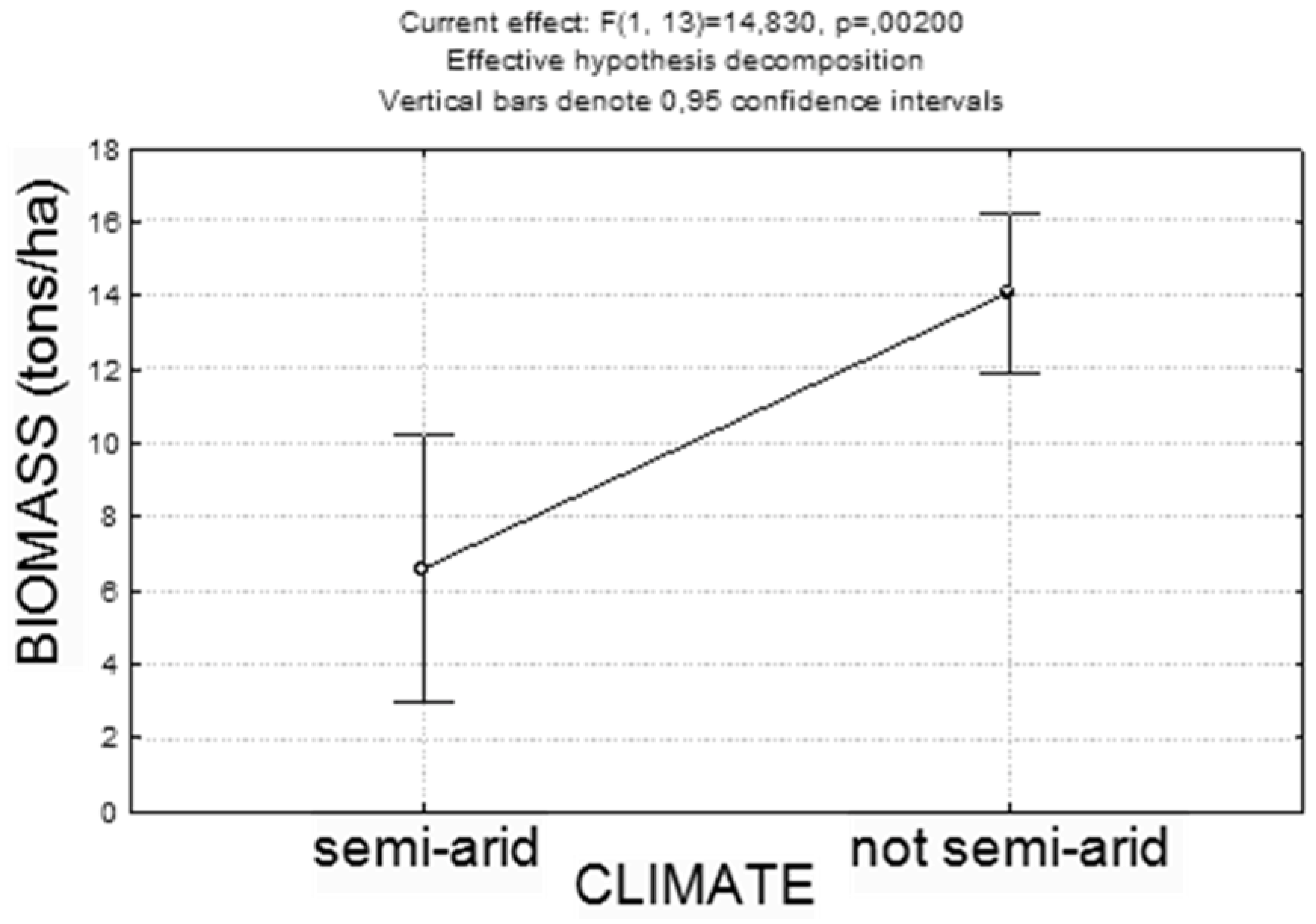

Supplement: Figure S6 — Relation between climate and biomass (tons/ha). (TIFF) [file pone.0085127.s006.tiff]

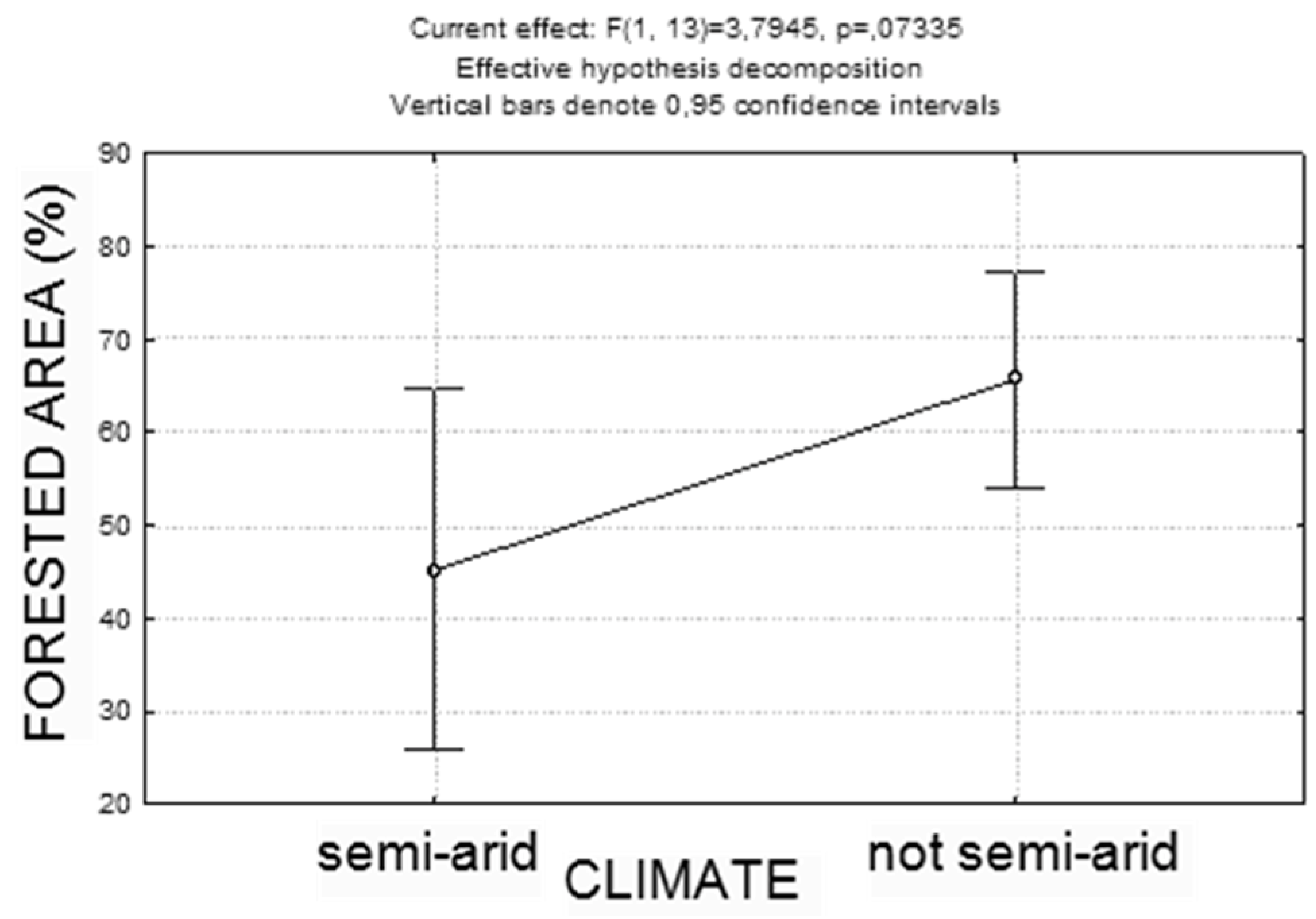

Supplement: Figure S7 — Relation between climate and forested area (%). (TIF) [file pone.0085127.s007.tiff]
