# Supplementary material for: Extreme Fire Severity Patterns in Topographic, Convective and Wind-Driven Historical Wildfires of Mediterranean Pine Forests
Source: PLoS One. 2014 Jan 22;9(1):e85127. doi: 10.1371/journal.pone.0085127 (PMC3899010; doi:10.1371/journal.pone.0085127)
Supplement: Table S1 — Information about the fires analyzed. Fire name, date of ignition, path/row, pre-fire data, post-fire data, sensor of the images, fire size, range of elevation, range of slope and fire type. (PDF) [file pone.0085127.s008.pdf]

Table S1. Information about the fires analyzed. Fire name, date of ignition, path/row, pre-fire data, post-fire data, sensor of the images, fire size, range of elevation, range of slope and fire type.

Fire name	Date of ignition	path/row	Pre-fire image data	Post-fire image data	Sensor	Fire size (ha)	Elevation (m)	Slope (°)	Fire type
Bot 2000	13-Aug-2000	198/30;198/31	14-jul-99	04-oct-00	ETM+	258	244-593	0-56	T
Castellbisbal 2002	11-jul-02	198/30	28-jun-02	22-jul-02	TM/ETM+	148	58-170	0.5-43	T
Castellbell i el Vilar 2003	13-jul-03	198/30;197/31	01-jul-03	26-jul-03	TM	371	166-363	0-48	T
Talamanca 2003	19-jul-03	197/31	10-jul-03	26-jul-03	TM	162	420-612	0.5-44	T
Castellbisbal 2005	05-jul-05	197/31	29-jun-05	15-jul-05	TM	206	46-187	0-51	T
Vimbodí 2006	21-jun-06	198/31	20-jun-05	23-jun-06	TM	123	540-710	0.5-35	T
Navàs 2007	04-jul-07	198/31	26-jun-07	28-jul-07	TM	226	337-667	0.5-44	T
Margalef 2005	26-may-05	198/31	19-may-05	20-jun-05	TM	434	454-667	0-53	C
Rocafort 2005	19-jun-05	197/31;198/31	28-may-05	20-jun-05	TM	726	292-527	0-42	C
Cardona 2005	08-jul-05	198/31	06-jul-05	22-jul-05	TM	1438	359-742	0-47	C
Castellnou de Bages 2005	18-jul-05	197/31;198/31	15-jul-05	22-jul-05	TM	825	281-610	0-50	C
Riba-roja d'Ebre 2005	22-Aug-2005	198/31	07-Aug-05	23-Aug-05	TM	575	53-473	0.5-44	W
Cistella 2006	04-Aug-2006	197/31	18-jul-06	04-sep-06	TM	203	117-265	0-32	W
Ventalló 2006	04-Aug-2006	197/31	03-Aug-06	04-sep-06	TM	959	8-174	0-26	W
Mont-roig del Camp 2007	04-jul-07	198/31	23-jun-06	12-jul-07	TM	401	0-65	0-19	W

T: topographic fire; C: convection-dominated fire; W: wind-driven fire
