# Supplementary material for: Extreme Fire Severity Patterns in Topographic, Convective and Wind-Driven Historical Wildfires of Mediterranean Pine Forests
Source: PLoS One. 2014 Jan 22;9(1):e85127. doi: 10.1371/journal.pone.0085127 (PMC3899010; doi:10.1371/journal.pone.0085127)
Supplement: Table S2 — Bands related to Crown Biomass, regression type, B0(constant), B1(regression coefficient) and R square. (PDF) [file pone.0085127.s009.pdf]

Table S2. Bands related to Crown Biomass, regression type, B_0(constant), B_1(regression coefficient) and R square.

Band	regression type	B_0	B_1	R square
TM1	exponential	20.798	-0.114	0.182
TM2	exponential	19.677	-0.116	0.158
TM3	exponential	18.578	-0.106	0.223
TM4	exponential	17.927	-0.037	0.038
TM5	exponential	36.504	-0.086	0.303
TM7	exponential	23.093	-0.114	0.282
NDVI= (TM4-TM3)/ (TM4+TM3)	exponential	1.086	3.985	0.237
NDMI= (TM4-TM5)/ (TM4+TM5)	exponential	1.388	4.796	0.295
Ratio TM4/TM3	exponential	2.154	0.409	0.217
Ratio TM5/TM4	logarithmic	6.999	-18.954	0.252
Ratio TM7/TM4	logarithmic	-0.655	-13.892	0.306
MID57= TM5+TM7	exponential	33.393	-0.052	0.336
TC1 (Brightness)	exponential	29.817	-0.044	0.163
TC2 (Greenness)	exponential	3.089	0.096	0.146
TC3 (Wetness)	exponential	1.13	0.102	0.311
