# Supplementary material for: Extreme Fire Severity Patterns in Topographic, Convective and Wind-Driven Historical Wildfires of Mediterranean Pine Forests
Source: PLoS One. 2014 Jan 22;9(1):e85127. doi: 10.1371/journal.pone.0085127 (PMC3899010; doi:10.1371/journal.pone.0085127)
Supplement: Table S3 — Parameter estimates of the regression between Crown Biomass and MID57 index. (PDF) [file pone.0085127.s010.pdf]

Table S3. Parameter estimates of the regression between Crown Biomass and MID57 index.

Equation	Model Summary					Parameter Estimates	
	R Square	F	df1	df2	Sig.	Constant	b1
Exponential	0.400	183.158	1	275	0.000	42.177	-0.061
